# ZIF-8-Embedded Cation-Exchange Membranes with Improved Monovalent Ion Selectivity for Capacitive Deionization

**DOI:** 10.3390/membranes15010019

**Published:** 2025-01-09

**Authors:** Eui-Gyu Han, Ji-Hyeon Lee, Moon-Sung Kang

**Affiliations:** Department of Green Chemical Engineering, College of Engineering, Sangmyung University, Cheonan 31066, Republic of Korea; 2022D2008@sangmyung.kr (E.-G.H.); 2021D3009@sangmyung.kr (J.-H.L.)

**Keywords:** membrane capacitive deionization, cation exchange membrane, metal–organic framework, ZIF-8, sieving effect, permselectivity for monovalent ions

## Abstract

Membrane capacitive deionization (MCDI) is an electrochemical ion separation process that combines ion-exchange membranes (IEMs) with porous carbon electrodes to enhance desalination efficiency and address the limitations of conventional capacitive deionization (CDI). In this study, a cation-exchange membrane (CEM) embedded with a metal–organic framework (MOF) was developed to effectively separate monovalent and multivalent cations in influent solutions via MCDI. To fabricate CEMs with high monovalent ion selectivity, ZIF-8 was incorporated into sulfonated poly(2,6-dimethyl-1,4-phenylene oxide) (SPPO) at various weight ratios. The resulting membranes were systematically characterized using diverse electrochemical methods. The ZIF-8-embedded CEMs demonstrated a sieving effect based on differences in ion size and hydration energy, achieving excellent permselectivity for monovalent ions. MCDI tests using the prepared CEMs showed a Na^+^ ion removal rate exceeding 99% in Na^+^/Mg^2+^ and Na^+^/Ca^2+^ mixed feed solutions, outperforming a commercial membrane (CSE, Astom Corp., Tokyo, Japan), which achieved a removal rate of 94.1%. These findings are expected to provide valuable insights for advancing not only MCDI but also other electro-membrane processes capable of selectively separating specific ions.

## 1. Introduction

Only about 0.5% of the water on Earth is freshwater, while the remaining 98% is seawater. As a result, the scarcity of freshwater has become a critical issue for humanity, especially with the rapid growth of the global population. Furthermore, the water shortage crisis is being exacerbated by worsening environmental pollution [1,2,3,4,5]. Consequently, there is growing global interest in desalination technologies, and related research is being actively pursued. Promising desalination methods beyond conventional distillation, which requires high energy consumption, include reverse osmosis (RO), electrodialysis (ED), and capacitive deionization (CDI). Among these, CDI has garnered significant attention as a cost-effective and environmentally friendly desalination technology due to its low energy requirements and lack of secondary pollution [5,6,7,8,9].

**Figure 1:**
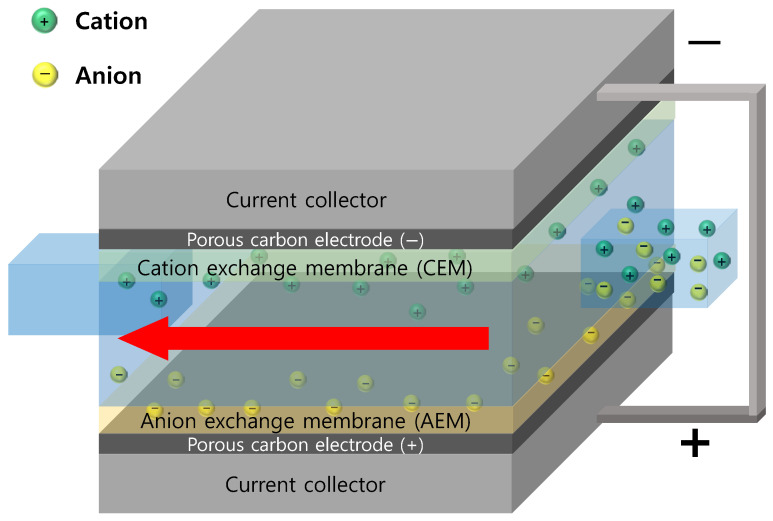
Schematic drawing of the MCDI operation principle.

The CDI process is an electrochemical desalination technology that adsorbs cations and anions from water onto the surface of porous carbon electrodes by applying an electrical potential. Additionally, by switching the electrode polarity or turning off the power, the adsorbed ions can be released back into the water, regenerating the electrodes. However, if ionic substances are not completely removed during this process, the electrodes are only partially regenerated, leading to a reduced adsorption capacity. To address these limitations and enhance desalination efficiency, membrane capacitive deionization (MCDI)—which integrates ion-exchange membranes (IEMs) with porous carbon electrodes—has been actively researched [10,11,12,13]. In MCDI, ions are selectively adsorbed after being separated by an IEM in close contact with the carbon electrodes. This configuration effectively suppresses the undesired adsorption of oppositely charged ions onto the electrodes during the desorption process. As a result, MCDI achieves more efficient ion separation compared to CDI, improving its overall desalination performance. Figure 1 illustrates a schematic diagram of the structure and operating principle of MCDI [14].

Recently, separation processes that selectively remove specific ions from influent solutions containing various ionic species, such as seawater, have garnered significant interest [9,11,12,13,15,16]. For instance, separating monovalent from multivalent ions is often necessary to enhance the efficiency of subsequent processes that utilize treated water. Efficient separation of specific ions is also critical for purposes such as removing hazardous ionic substances from wastewater or concentrating target ions. CDI has gained attention as an efficient method for the selective desalination and concentration of specific ions [9]. In CDI, selective ion separation can be achieved by controlling the adsorptive properties of the electrode or by employing an IEM with selective permeability for specific ions. To date, most research has focused on modifying electrode properties to achieve selective ion separation [9]. Meanwhile, the development of IEMs designed to selectively separate specific ions in various electro-membrane processes has been actively pursued. As a result, the application of MCDI, which leverages the selective separation characteristics of IEMs, is also receiving growing attention.

The selective ion separation characteristics of IEMs are generally determined by electrostatic repulsion and steric hindrance effects. Electrostatic repulsion enables the separation of ions with different valences by controlling the strength and polarity of fixed charges on the membrane surface. For instance, if a positively charged layer is introduced on the surface of a cation-exchange membrane (CEM), monovalent cations can pass through, whereas divalent cations are blocked due to stronger electrostatic repulsion. Furthermore, by adjusting the degree of cross-linking in the membrane, ions with different hydration radii can be separated through the steric hindrance effect [17,18].

Metal–organic frameworks (MOFs), porous crystalline materials in which metal ions and organic molecules are linked by organic ligands to form three-dimensional skeletal structures, have recently been actively used as additives to improve the selective permeability of membranes [19,20,21]. MOFs can be synthesized into a wide variety of structures depending on the metal and organic components used, with various functionalizations possible when highly reactive organic components are included. Due to their unique features, such as diverse structures, controllable pore sizes, and modifiable frameworks, MOFs have been widely applied in separation, catalysis, electrochemistry, and biomedical fields [22,23,24,25,26,27,28,29,30,31]. Among the many types of MOFs, zeolitic imidazolate frameworks (ZIFs) offer advantages such as simple synthesis, low cost, and high specific surface area. In particular, ZIF-8, composed of dimethylimidazoles and zinc ions (Zn^2+^), exhibits strong hydrophobicity, and its pore window size (3.4 Å) is slightly smaller than the hydration radii of both monovalent and multivalent ions.

In this study, composite CEMs were fabricated by incorporating ZIF-8 nanoparticles as fillers into a sulfonated poly(2,6-dimethyl-1,4-phenylene oxide) (SPPO) matrix and applied to MCDI for the selective separation of monovalent cations. The well-developed pore structure and hydrophobicity of ZIF-8’s ion diffusion channels were expected to effectively separate monovalent and multivalent ions based on differences in ion size and hydration energy. To determine the optimal composition for maximizing monovalent ion selectivity and electrochemical properties, CEMs were prepared with varying amounts of ZIF-8, and their properties were systematically evaluated using various electrochemical analyses. Furthermore, the selective desalination performance was assessed by comparing commercial and fabricated membranes in MCDI applications. The findings of this study are expected to provide valuable insights for the development of IEMs and MCDI processes, not only for the separation of monovalent ions but also for the selective separation of other specific ions.

## 2. Experimental

### 2.1. ZIF-8 Synthesis and Characterizations

To synthesize ZIF-8 nanoparticles, 8 g of zinc nitrate hexahydrate and 3.6 g of 2-methylimidazole were each completely dissolved in 200 mL of methanol (MeOH) by stirring for 30 min. The two solutions were then mixed and allowed to react for 1 h. After the reaction, the mixed solution was centrifuged at 3500 rpm to precipitate the ZIF-8 nanoparticles, which were subsequently separated from the solvent. The resulting precipitate was dried in an oven (OF-12GW, JEIO TECH, Daejeon, Republic of Korea) at 120 °C for over 12 h to obtain a white powder [32,33]. The morphological characteristics of the synthesized ZIF-8 nanoparticles were examined using a field emission-scanning electron microscope (FE-SEM, SIGMA500, Carl ZEISS, Jena, Germany). Additionally, Fourier transform infrared spectroscopy (FT-IR, FT/IR-4700, Jasco, Hachioji, Japan) and X-ray diffraction (XRD, MiniFlex 600, Rigaku, Tokyo, Japan) analyses were conducted to confirm the chemical and crystal structures of the ZIF-8 nanoparticles, respectively.

### 2.2. Membrane Fabrication and Characterizations

To prepare SPPO, 2.75 g (23.4 mmol) of chlorosulfonic acid (CSA) was first dissolved in 8.25 mL of chloroform. Subsequently, 10 g of PPO was dissolved in 108.25 mL of chloroform to prepare a PPO solution, to which the CSA solution was slowly added dropwise while stirring vigorously. The PPO/CSA molar ratio of the mixed solution was maintained at 0.351. After the reaction was complete, the mixed solution was added dropwise to distilled water to obtain a solid polymer, which was then washed more than five times with distilled water and dried in an oven at 60 °C for over 12 h to prepare SPPO.

A casting solution was prepared by dissolving SPPO in *N*,*N*-dimethylacetamide (DMAc) at 25 wt% for fabricating the composite CEMs. The prepared ZIF-8 nanoparticles were then added to the casting solution at concentrations of 0, 1, 2.5, 5, 7.5, 10, 12.5, and 15 wt%, respectively. The nanoparticles were evenly dispersed in the solution via sonication and stirred at 60 °C for 24 h. The resulting SPPO-ZIF-8 solution was cast onto a glass plate and dried at room temperature for 4 h, followed by drying at 60 °C for 24 h, to fabricate the composite CEMs.

All reagents used in the experiments were purchased from Sigma-Aldrich Corp. (St. Louis, MO, USA) and used as received. To compare the characteristics of the prepared membranes, a commercial membrane, Neosepta CSE (Astom Corp., Tokyo, Japan), was used as a reference membrane. Neosepta CSE is a standard-grade commercial membrane known for its excellent electrochemical properties and high reliability. It is widely used in electo-membrane processes for ion separation, making it a suitable reference for comparing the basic performance of the fabricated CEMs.

To confirm the uniform dispersion of nanomaterials within the fabricated composite CEM, the membrane cross-section was observed at a magnification of 10,000× using a field-emission scanning electron microscope (FE-SEM, GeminiSEM 560, Carl Zeiss, Jena, Germany) and energy-dispersive X-ray spectroscopy (EDS, Ultim Max, Oxford Instruments, Abingdon, UK) analysis was also performed”.

Additionally, to measure the electrical resistance (ER) of the membranes, the CEM samples were immersed in a 0.5 M NaCl solution for over 6 h to reach equilibrium with the solution. The impedance of the membranes was then measured in 0.5 M NaCl solution using a lab-made clip cell connected to a potentiostat/galvanostat (SP-150, Bio-Logic Science Instruments, Grenoble, France), and the ER values were calculated using Equation (1):

(1) $$ER=\left({\left|Z\right|}_{sample}\times {\cos\theta }_{sample}-{\left|Z\right|}_{blank}\times {\cos\theta }_{blank}\right)\times A$$

where ${\left|Z\right|}_{sample}$ is the impedance of the electrolyte and membrane (Ω), ${\left|Z\right|}_{blank}$ is the impedance of the electrolyte (Ω), *θ* is the phase angle (degree), and *A* is the membrane area (cm^2^). The ion conductivity of the membranes was calculated by dividing the ER value by the membrane thickness and taking the reciprocal.

The ion transport number (TN, $t_{+}$) of the membranes was determined by substituting the membrane potential measured using a pair of Ag/AgCl reference electrodes in a lab-made two-compartment cell into Equation (2) [32,33]:

(2) $$E_{m}=\frac{RT}{F}({2t}_{+}-1)\ln\frac{a_{L}}{a_{H}}$$

where *E_m_* is the measured membrane potential (mV), *R* is the gas constant, *T* is the absolute temperature, *F* is the Faraday constant, and $a_{L}$ and $a_{H}$ are the concentrations of NaCl solution (1 mM and 5 mM, respectively).

The ion-exchange capacity (IEC) of the membranes was measured using traditional acid-base titration. First, the membrane sample was immersed in a 0.5 M HCl solution for over 6 h to replace all functional groups with H^+^ ions. The acid solution on the membrane surface was then washed with distilled water, and the remaining water was removed using filter paper. Next, the membrane was immersed in a 0.5 M NaCl solution to re-substitute the H^+^ ions with Na^+^ ions. The amount of H^+^ ions in the solution was measured through acid-base titration using 0.01 M NaOH as a titrant and phenolphthalein as the indicator. After the titration was complete, the titration values and the dry weight of the membrane were substituted into Equation (3) to calculate the IEC:

(3) $$IEC\left(\frac{meq}{g}\right)=\frac{C_{H^{+},sample}\times V_{sample}}{W_{dry}}$$

where *V_sample_* is the volume (mL) of the titrant, *C_H+, sample_* is the concentration of the titrant (mol/L), and *W_dry_* is the dry weight (g) of the membrane used in the experiment.

The water uptake (WU) of the membrane was determined by measuring the wet weight (*W_wet_*) and dry weight (*W_dry_*) of the membrane, respectively, and substituting them into Equation (4).

(4) $$WU=\frac{W_{wet}-W_{dry}}{W_{dry}}\times 100\%$$

**Figure 2:**
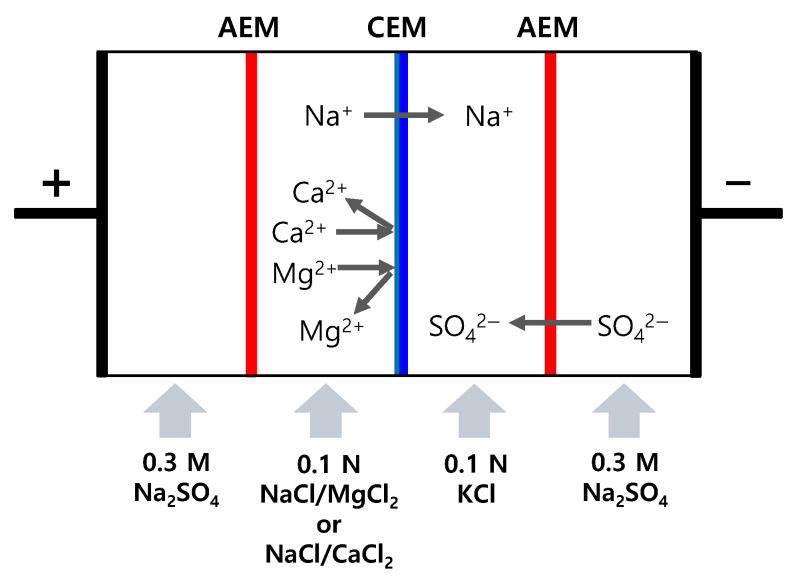
Schematic drawing of permselectivity measurement using 4-compartment electrodialysis cell.

The permselectivity (*P*) of the membrane was determined through electrodialysis experiments using a four-compartment cell as shown in Figure 2. For this, 200 mL of 0.1 M NaCl/MgCl_2_ or 0.1 M NaCl/CaCl_2_ was used as the feed solution, and 200 mL of 0.1 M KCl was filled and circulated in the permeate compartment. Additionally, 0.3 M Na_2_SO_4_ was circulated in the electrode compartment. The effective area of the Pt/Ti electrodes and the membrane were each 15 cm^2^, and the experiment was conducted for 1 h under a constant current density of 3.54 mA/cm^2^. The concentrations of Na^+^ and Mg^2+^ ions in the permeate compartment were measured using ion chromatography (883 Basic IC plus, Metrohm, Herisau, Switzerland), and the ion flux was calculated by substituting them into Equation (5):

(5) $$J=\frac{\left(C_{f}-C_{i}\right)}{A\times t}\times V$$

where *C_i_* and *C_f_* are the initial and final ion concentrations (mol/L), respectively, *A* is the effective area (cm^2^), *t* is the experiment time (h), and *V* is the volume (L). In addition, the permselectivity was calculated by substituting the ion flux of the two compared ions (*A*^+^ and *B*^2+^), obtained using the above formula, into Equation (6) [17].

(6) $$P_{B^{2+}}^{A^{+}}=\frac{J_{A^{+}}}{J_{B^{2+}}}$$

### 2.3. MCDI Cell Tests for Desalination

**Figure 3:**
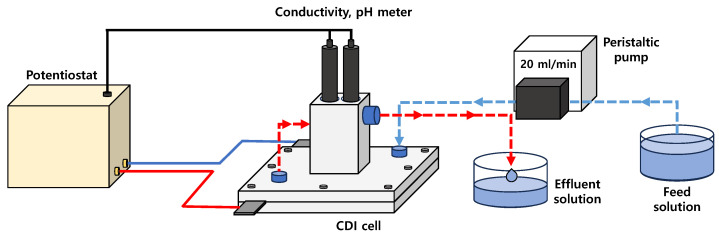
Schematic diagram showing MCDI process operation (red and blue solid lines: anode and cathode, respectively, red and blue dashed lines: effluent and influent, respectively).

Graphite electrodes laminated with porous activated carbon (effective area = 10 cm × 10 cm) were supplied by Siontech (Daejeon, Republic of Korea), and Neosepta ASE (Astom Corp., Tokyo, Japan) was used as the anion-exchange membrane (AEM). The effective area of the membrane was 11 cm × 11 cm, the thickness of the spacer was 2 mm, and the solution was delivered at a flow rate of 20 mL/min using a peristaltic pump. For the CDI experiment, a constant voltage of +1.5 V (for desalination) and 0 V (for regeneration) was applied, and the adsorption and desorption periods were 3 min each. A 1.7 × 10^−3^ M NaCl/MgCl_2_ or NaCl/CaCl_2_ solution was used as the influent. During the experiment, the influent and effluent concentrations were measured and substituted into Equation (7) to calculate the salt removal efficiency (*η*):

(7) $$\eta =\frac{C_{i}-C_{f}}{C_{i}}\times 100\%$$

where $C_{i}$ is the ion concentration of the influent and $C_{f}$ is the ion concentration of the effluent. Figure 3 displays a schematic diagram explaining the operation of the MCDI process.

## 3. Results and Discussion

**Figure 4:**
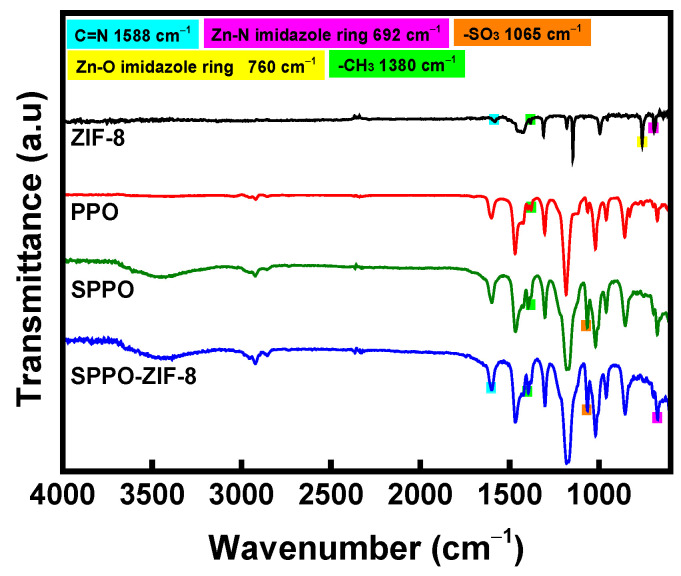
FT-IR spectra of ZIF-8, PPO, SPPO, and SPPO-ZIF-8 membrane.

FT-IR spectra were measured to confirm the sulfonation of PPO and the synthesis of ZIF-8, with the results shown in Figure 4. The peak at 1065 cm^−1^ in the SPPO spectrum corresponds to the asymmetric stretching of -SO_3_, which is an absorption peak not present in PPO, indicating that the sulfonic acid group (-SO_3_H) was successfully introduced into PPO [34]. Meanwhile, the chemical structure of ZIF-8 was confirmed by observing the absorption peaks at 1380 cm^−1^ and 1588 cm^−1^, which are assigned to the methyl group of 2-methylimidazole and the C=N bond in the imidazolium ring, respectively, in the ZIF-8 spectrum. The absorption peaks corresponding to Zn-O and Zn-N at 1588 cm^−1^ and 692 cm^−1^, respectively, were also found in the spectrum. Additionally, absorption peaks corresponding to ZIF-8 were observed in the spectrum of the SPPO-ZIF-8 membrane, demonstrating the successful preparation of the composite CEM [25,35,36].

**Figure 5:**
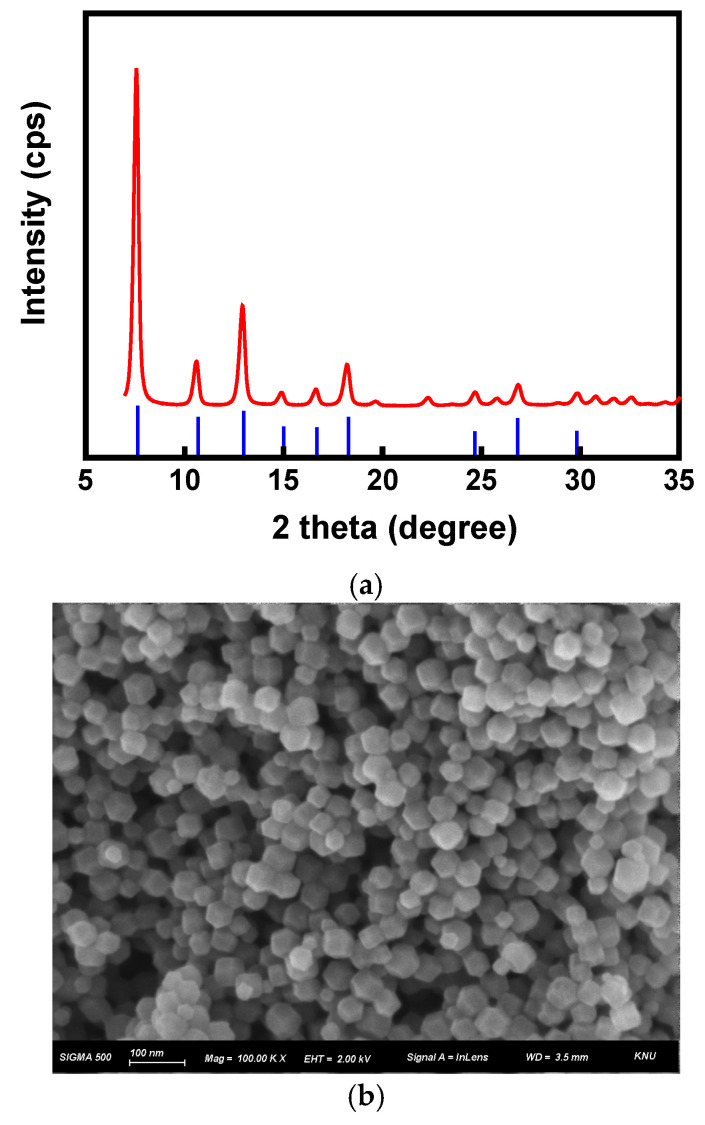
(**a**) XRD spectrum (red: ZIF-8 (measured) and blue: JCPDS 00-066-0091) and (**b**) FE-SEM image of ZIF-8 nanomaterial.

XRD analysis was performed to investigate the crystal structure of the synthesized ZIF-8 material. As shown in Figure 5a, the XRD pattern of the synthesized ZIF-8 displayed characteristic diffraction peaks at 2*θ* = 7.6°, 10.6°, 12.9°, 14.9°, 16.6°, 18.2°, 22.3°, 24.7°, 26.9°, and 29.8°, which were consistent with the reference (JCPDS 00-066-0091), confirming the typical sodalite structure of ZIF-8 [37,38]. Additionally, the FE-SEM image (Figure 5b) confirmed that the synthesized ZIF-8 nanoparticles had an approximate size of 30 nm and a uniform rhombic dodecahedral structure with a well-defined crystal surface, which is consistent with the XRD results [39].

**Figure 6:**
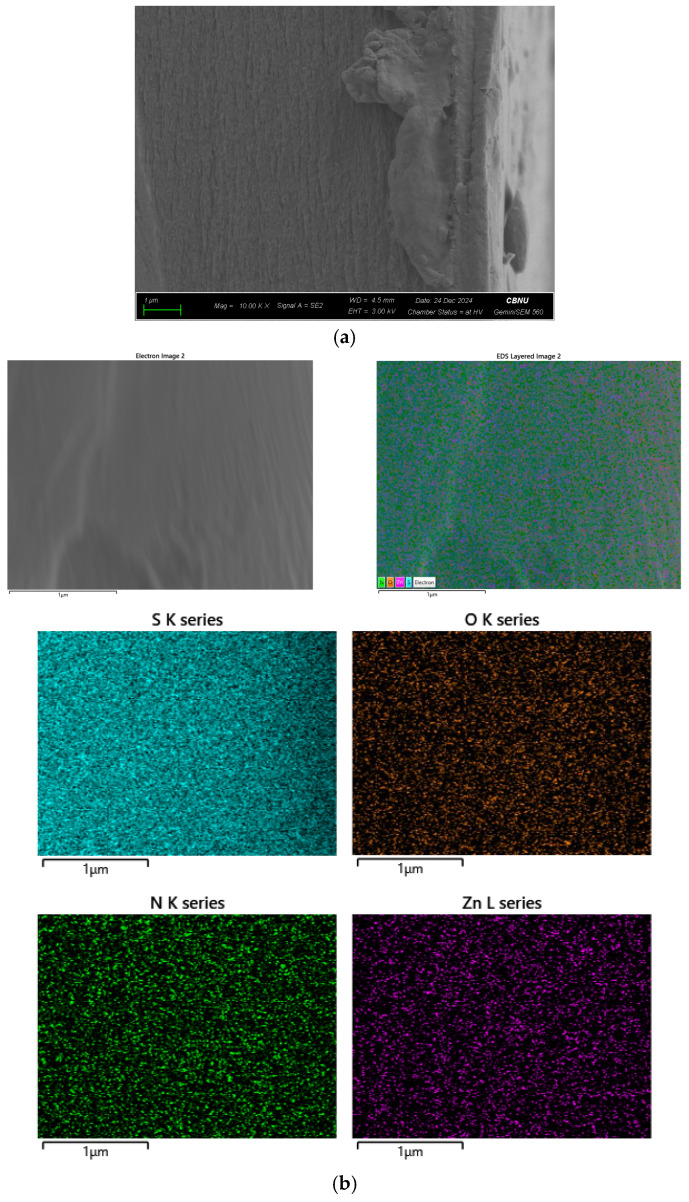
(**a**) FE-SEM image and (**b**) EDS mapping of SPPO-ZIF-8(2.5 wt%) membrane (cross-sectional).

To verify the even distribution of ZIF-8 throughout the thickness of the composite membrane, the cross-sectional image and EDS mapping results of the SPPO-ZIF-8 (2.5 wt%) membrane are presented in Figure 6. The results show that the S and O elements, associated with the sulfonic acid groups, were homogeneously distributed across the membrane’s cross-section [40]. Furthermore, the Zn and N elements originating from ZIF-8 were uniformly dispersed throughout the membrane thickness, confirming the even distribution of ZIF-8 nanoparticles within the composite CEM [41,42].

The results of the characterization of membranes fabricated with different ZIF-8 contents, along with the commercial membrane (Neosepta CSE), are summarized in Table 1. The thickness of the fabricated membranes was approximately 36 μm, which is much thinner than that of the CSE (ca. 143 μm). It was observed that as the ZIF-8 content in the prepared membranes increased, the ER value gradually rose, while the conductivity decreased. This indicates that as the content of ZIF-8 (which lacks ion-exchange groups) increases, ion transport through the membrane is somewhat hindered. However, due to the relatively thin membrane thickness compared to the commercial membrane, even at the maximum content of 15 wt%, the ER remained much lower than that of the CSE. The TN value of the fabricated membranes was approximately 0.97 across the range of ZIF-8 content used in the experiment, which was nearly equivalent to that of the commercial CSE membrane. The prepared membranes also exhibited an IEC value ranging from 1.99 to 1.52 meq./g, showing a decreasing trend as the ZIF-8 content increased. Meanwhile, the decrease in WU as the ZIF-8 content increased is thought to result from the increased hydrophobicity inside the membrane due to the methyl group contained in ZIF-8, along with the decrease in IEC.

**Figure 7:**
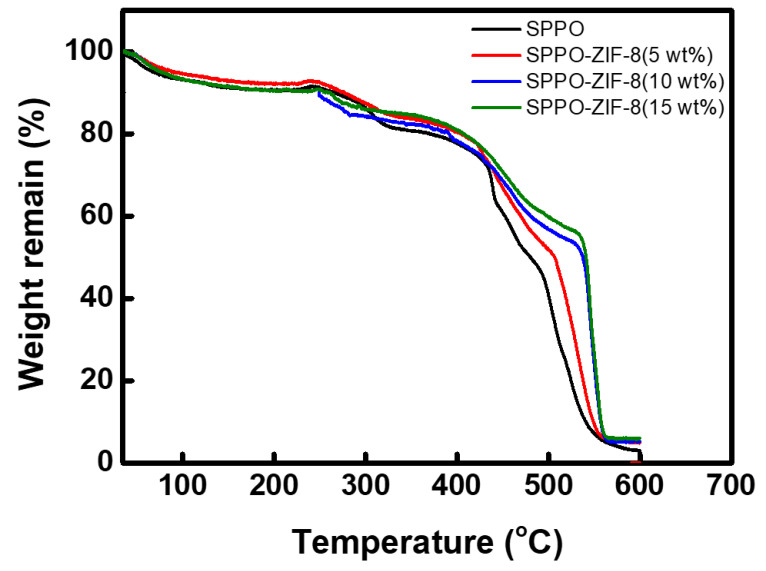
TGA thermograms of SPPO and SPPO-ZIF-8 membranes.

The thermal properties of the fabricated CEMs were evaluated through TGA analysis, and the results are shown in Figure 7. The thermograms of the SPPO and SPPO-ZIF-8 membranes exhibited three major stages of mass loss. Up to about 200 °C, mass loss occurred due to the removal of water and residual solvent absorbed into the membrane, and decomposition of the sulfonic acid groups occurred in the range of 200–300 °C. Additionally, above 400 °C, the greatest mass loss occurred due to the decomposition of the polymer backbone [43]. In the case of ZIF-8, oxidative decomposition of 2-methylimidazole is known to occur between 100 and 300 °C, and the decomposition of the organic linker in ZIF-8 crystals appears above approximately 550 °C [44]. When the ZIF-8 content was 10 wt% or higher, it was confirmed that approximately 50% or more of the membrane mass remained at around 550 °C due to the strong bonding of the ZIF-8 crystals.

**Figure 8:**
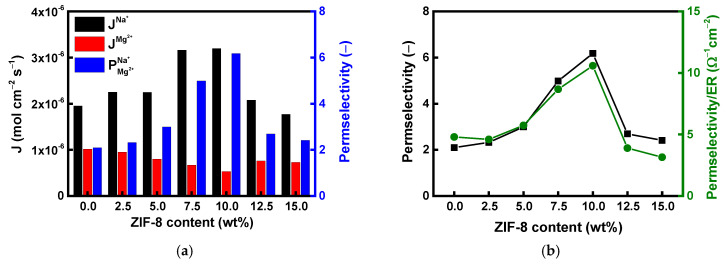

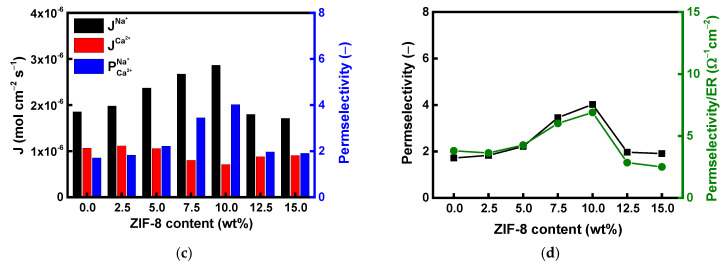
Ion flux, permselectivity, and permselectivity/ER ratio through composite CEMs fabricated with various ZIF-8 contents: (**a**,**b**) in Na^+^/Mg^2+^ solution and (**c**,**d**) in Na^+^/Ca^2+^ solution.

**Figure 9:**
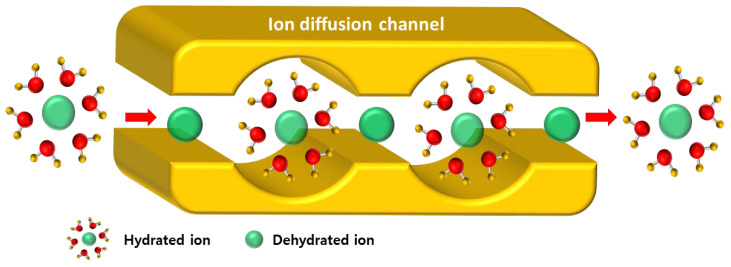
Schematic image showing selective transport mechanism by dehydration-hydration of ions through ion diffusion channel of ZIF-8.

Figure 8 shows the ion flux, permselectivity, and permselectivity/ER ratio measured in Na^+^/Mg^2+^ and Na^+^/Ca^2+^ mixed solutions, highlighting the monovalent ion selective permeation characteristics of SPPO membranes with varying ZIF-8 contents. The results revealed that as the ZIF-8 content increased up to 10 wt%, the Na^+^ flux increased and then decreased significantly. On the other hand, for Mg^2+^ and Ca^2+^, the flux decreased as the ZIF-8 content increased up to 10 wt%, followed by a slight increase in flux. As a result, under both solution conditions, the highest permselectivity for monovalent ions was observed at 10 wt% ZIF-8 content, with permselectivity values exceeding 6 for Mg^2+^ and 4 for Ca^2+^ ions, respectively. A schematic illustrating the effect of ZIF-8 on improving the permselectivity for monovalent ions is shown in Figure 9. The basic ion separation mechanism of CEMs is based on the electrostatic attraction of counterions and the repulsion of coions by ion exchange groups (i.e., sulfonic acid groups, -SO_3_^−^) within the membranes, according to traditional theory. Meanwhile, ZIF-8 does not contain ion exchange groups but can act as a selective barrier, enhancing the monovalent ion selectivity of CEMs by controlling the transport of monovalent and divalent ions. ZIF-8 is dispersed in the SPPO matrix to form a hydrophobic ion diffusion channel, through which hydrated ions must pass via a dehydration-hydration process [45,46,47,48,49,50]. In general, monovalent ions have lower hydration energies than divalent ions, making it relatively easier for monovalent ions to pass through the ion diffusion channels of ZIF-8, allowing for selective separation through the membrane. The hydration radii of Na^+^, Ca^2+^, and Mg^2+^ are 3.58, 4.12, and 4.28 Å, respectively, and their hydration energies are −310, −1481, and −1752 kJ/mol, respectively. Therefore, Na^+^ ions, which have a relatively small hydration radius and are easier to dehydrate and rehydrate, can pass through the ion diffusion channels of ZIF-8 more easily than Ca^2+^ and Mg^2+^ ions [30,50,51]. Based on this principle, the flux and permselectivity for Na^+^ ions are expected to increase as the amount of ZIF-8 added to SPPO increases. When comparing Ca^2+^ and Mg^2+^ ions under the same membrane conditions, it was also observed that the permselectivity for Mg^2+^ ions, which have a relatively larger hydration radius and hydration energy, was higher than for Ca^2+^ ions. However, as shown in Table 1, when an excessive amount of ZIF-8 was added (greater than 12.5 wt% in this study), the ER of the membrane increased significantly due to the aggregation of ZIF-8 particles and the decrease in IEC. It is well known that in mixed-matrix membranes (MMMs), exceeding the optimal filler content often leads to undesired particle aggregation, which can obstruct the formation of effective mass transfer channels [52]. It was believed that the ion separation through the dehydration-hydration process was hindered in this situation, leading to a decrease in permselectivity for monovalent ions. Additionally, the ratio of permselectivity to ER was calculated, revealing the same trend as permselectivity, with the optimal membrane production conditions determined at 10 wt% ZIF-8 content.

**Figure 10:**
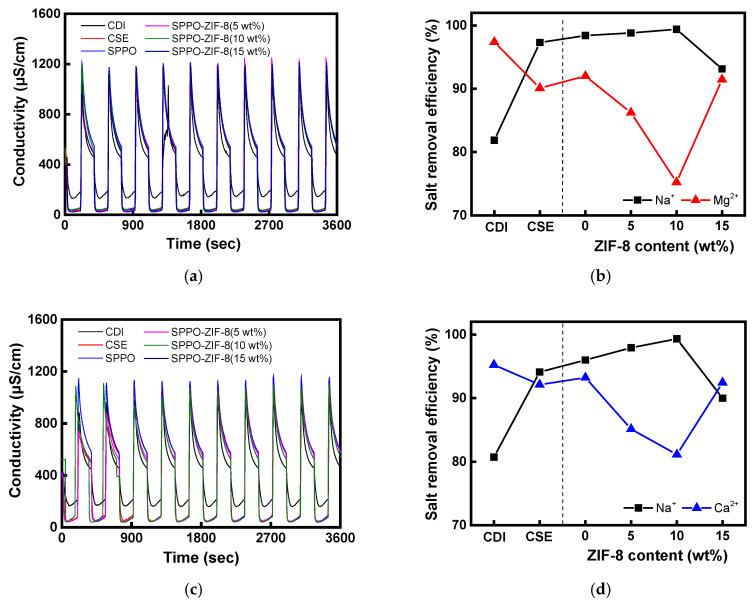
Desalination performances of CDI and MCDI: (**a**) time-course change in conductivity; (**b**) salt removal efficiency for Na^+^/Mg^2+^ feed solution and (**c**) time-course change in conductivity; (**d**) salt removal efficiency for Na^+^/Ca^2+^ feed solution.

Based on the results of the previous selectivity evaluation, MCDI experiments were performed using commercial membranes and composite CEMs fabricated with 0, 5, 10, and 15 wt% ZIF-8 contents, and the results are shown in Figure 10. In this experiment, the performance of CDI without a membrane was also assessed for comparison. After checking the conductivity changes over 10 cycles (3 min for adsorption/3 min for desorption) over the course of 1 h, it was confirmed that CDI exhibited significantly lower adsorption and desorption performance compared to MCDI. In CDI, when an ionic substance is adsorbed onto the electrode surface, and a reverse potential is applied, the ions adsorbed on the electrode are desorbed and move into the bulk solution, while the counter ions move from the solution to the electrode. This prevents complete desorption of the ionic substances from the electrode, resulting in incomplete regeneration. On the other hand, the MCDI process has high salt removal efficiency by preventing desorbed ions from being re-adsorbed onto the counter electrode by the IEM [53]. In the case of CDI, the removal rates of Mg^2+^ and Ca^2+^ were found to be 98% and 95%, respectively, under Na^+^/Mg^2+^ and Na^+^/Ca^2+^ feed conditions, while the removal rate of Na^+^ was approximately 80%. This is because ions with higher valence have a stronger interaction with the charged electrode surface, leading to increased adsorption to the electrical double layer (EDL). Therefore, when treating influent containing a mixture of monovalent and divalent ions, divalent ions are more likely to be adsorbed onto the electrode surface than monovalent ions at equilibrium, due to their stronger electrostatic attraction [54]. In the case of MCDI, the removal rate of monovalent cations was measured to be much higher than that of divalent cations. This is because the IEM used with the electrode has a higher permselectivity for monovalent ions than for divalent ions. Moreover, the SPPO-ZIF-8 composite CEM fabricated with 10 wt% ZIF-8 content among the comparative membranes exhibited the highest selective removal efficiency for monovalent ions in the CDI test. Under both Na^+^/Mg^2+^ and Na^+^/Ca^2+^ mixed solution conditions, the Na^+^ removal efficiency exceeded 99%, while the Mg^2+^ and Ca^2+^ removal rates showed the lowest values among the comparative membranes. This is interpreted as a result of using a monovalent ion-selective CEM in combination with an electrode, which prevents divalent ions from passing through the membrane during adsorption and causes them to remain in the influent, resulting in a higher removal rate of Na^+^ compared to divalent ions. However, when the SPPO-ZIF-8(15 wt%) membrane was applied to CDI, it showed a significantly lower monovalent ion removal rate than the other membranes under both the Na^+^/Mg^2+^ and Na^+^/Ca^2+^ mixed solution conditions, which corresponds to the lower permselectivity described above.

## 4. Conclusions

In this study, composite CEMs with excellent monovalent ion selectivity were prepared by embedding ZIF-8 nanoparticles in an SPPO matrix at various weight ratios for MCDI applications. ZIF-8 does not have ion-exchange capabilities and has high hydrophobicity, which somewhat reduces the ion conductivity of the composite membranes. However, the membrane resistance was lower than that of the commercial membrane due to the uniform dispersion of ZIF-8 nanoparticles and the thin membrane thickness. As a result of measuring the permselectivity for monovalent ions, the highest value was observed at 10 wt% ZIF-8 content, with permselectivity for monovalent ions greater than 6 and 4 for Na^+^/Mg^2+^ and Na^+^/Ca^2+^, respectively. In addition, CDI experiments were performed using the fabricated SPPO-ZIF-8 composite CEMs, and the results revealed a high Na^+^ ion removal efficiency of over 99% in both Na^+^/Mg^2+^ and Na^+^/Ca^2+^ mixed solution conditions at 10 wt% ZIF-8 content. The significant improvement in monovalent ion selectivity is attributed to the pore structure and the hydrophobicity of the ion diffusion channels of ZIF-8 nanoparticles. It is believed that selective ion separation can be achieved by combining the unique characteristics of the ZIF-8 nanoparticles and the differences in the hydration radii and hydration energies of the monovalent and divalent ions. The results of this study are expected to provide useful information for the development of various electro-membrane processes, including MCDI, which can effectively separate specific ions as well as monovalent and multivalent ions.

## Figures and Tables

**Table 1:** Characteristics of membranes fabricated with commercial polymeric membranes and various ZIF-8 contents.

Membranes	ER (Ω·cm^2^)	Conductivity (mS/cm)	TN (−)	IEC (meq./g)	WU (%)
CSE (Astom, Tokyo, Japan)	1.87	7.65	0.983	2.20	29.80
SPPO-ZIF-8 (0 wt%)	0.431	8.50	0.966	1.99	28.20
SPPO-ZIF-8 (1 wt%)	0.502	7.24	0.970	1.98	28.04
SPPO-ZIF-8 (2.5 wt%)	0.504	7.06	0.968	1.96	27.82
SPPO-ZIF-8 (5 wt%)	0.521	6.87	0.971	1.89	26.94
SPPO-ZIF-8 (7.5 wt%)	0.574	6.31	0.962	1.91	27.60
SPPO-ZIF-8 (10 wt%)	0.584	6.03	0.973	1.88	26.88
SPPO-ZIF-8 (12.5 wt%)	0.691	5.41	0.969	1.64	24.10
SPPO-ZIF-8 (15 wt%)	0.764	4.84	0.967	1.52	23.34

## Data Availability

Data are contained within the article.
